# Outpatient management of essential hypertension: a review based on the latest clinical guidelines

**DOI:** 10.1080/07853890.2024.2338242

**Published:** 2024-04-11

**Authors:** Areesha Moiz, Tetiana Zolotarova, Mark J. Eisenberg

**Affiliations:** a Centre for Clinical Epidemiology, Lady Davis Institute, Jewish General Hospital, Montreal, Canada; b Department of Medicine and Health Sciences, McGill University, Montreal, Canada; c Departments of Epidemiology, Biostatistics and Occupational Health, McGill University, Montreal, Canada; d Division of Cardiology, Jewish General Hospital, McGill University, Montreal, Canada

**Keywords:** hypertension, essential hypertension, blood pressure, guidelines, pharmacotherapy

## Abstract

**Background: **Essential hypertension, a prevalent cardiovascular condition, poses a significant health burden worldwide. Based on the latest American clinical guidelines, half of adults in the United States have hypertension. Of these, only about a half are treated and about a quarter are adequately controlled for hypertension. Given its impact on morbidity and mortality, ensuring effective management of high blood pressure is crucial to reduce associated risks and improve patient outcomes.**Objective: **This review aims to provide a comprehensive and up-to-date summary of the latest cardiology guidelines and evidence-based research on essential hypertension, with a focus on guiding outpatient clinical practice.**Methods:** The review evaluates both non-pharmacological approaches and pharmacological interventions to offer clinicians practical insights. Notably, it emphasizes the importance of individualized treatment plans tailored to patients’ specific risk profiles and comorbidities.**Results:** By consolidating the latest advancements in hypertension management, this review provides clinicians with an up-to-date reference, offering a nuanced understanding of treatment goals and strategies.**Conclusion: **Through the incorporation of evidence-based recommendations, healthcare practitioners can optimize patient care, mitigate potential complications, and improve overall outcomes in essential hypertension.

## Introduction

Hypertension is one of the most common chronic medical conditions characterized by a persistent elevation in arterial blood pressure (BP) [1], which contributes to the development of stroke [2,3], myocardial infarction [4,5], heart failure [6], and renal failure [7]. In the United States, hypertension accounts for more cardiovascular disease (CVD)-related deaths than any other modifiable risk factor and comes in second to cigarette smoking as a preventable cause of death for any reason [8]. According to the latest diagnostic criteria as defined by the 2017 ACC/AHA guidelines [1], nearly half of American adults have hypertension [9]. Although approximately 80% of these adults are recommended prescription BP medication and lifestyle changes, 25% are adequately controlled for hypertension [9]. The prevalence of hypertension increases with age and is higher in men (50.4%) than women (44.3%), and in Africans Americans (56.2%) than any other ethnic group [9]. Additionally, there is a marked discrepancy between the high-income Western world and the world at large in terms of the diagnosis, treatment, and adequate control of hypertension [10]. This review aims to summarize the latest cardiology guidelines and evidence-based research to provide an overview of the current treatment goals and strategies for essential hypertension, and to guide outpatient clinical practice. The literature search was conducted using PubMed and Google Scholar with a focus on articles with high quality of evidence and low risk of bias. Relevant clinical guidelines, randomized controlled trials, systematic reviews or meta-analyses pertaining to essential hypertension were identified through title screening or snowballing references of included articles.

## Etiology

Hypertension can be divided into two major categories: essential (or primary) hypertension and secondary hypertension [1]. Essential hypertension, which does not have a single identifiable cause, represents the vast majority of cases (85–95%) [1,11]. Risk factors for essential hypertension include genetics, obesity, diabetes, smoking, high alcohol consumption, high sodium intake, lack of exercise, and stress [1]. In contrast, secondary hypertension is caused by an identifiable underlying condition which can be divided into four categories: renal, endocrine, vascular, or other [12]. Among these, renal causes include renal parenchymal disease or renovascular disease; endocrine causes are adrenal-dependent, thyroid-dependent, or pituitary dependent; vascular causes include coarctation of the aorta; and other causes include obstructive sleep apnea, drug-induced, and pregnancy. Since the prevalence of secondary hypertension is relatively low (5–15%), routine evaluations in the absence of compelling clinical findings are time-consuming and not cost-effective [13].

## Diagnostic criteria

In 2017, the American College of Cardiology in conjunction with the American Heart Association (ACC/AHA) released an updated document for the prevention, detection, evaluation, and management of high BP in adults [1]. Of note, this document lowered the diagnostic threshold of hypertension from the preceding 2003 Joint National Committee definition of 140/90 mmHg [14] to 130/80 mmHg (Table 1). This threshold was set irrespective of age and co-morbid illness status. Based on this new definition, the prevalence of hypertension increased by approximately 14% in the American population with an additional 4.2 million patients requiring antihypertensive treatment and 7.9 million patients requiring intensification of treatment [15]. In parallel, a year later, the European Society of Cardiology and European Society of Hypertension (ESC/ESH) updated and released their clinical practice guidelines, however, retaining the previous 140/90 mmHg diagnostic threshold (Table 1) [13]. The discrepancy regarding the diagnostic threshold between these two guidelines carried significant implications with respect to treatment initiation and therapeutic goals across populations. While the new American guidelines were seen as pragmatic, aiming to reduce hypertension-related disease burden by early risk detection and intervention, their European counterpart were more conservative with focus on the individual patient rather than epidemiological concerns [16]. New studies are required to elucidate which strategy has had a bigger impact on the reduction of cardiovascular morbidity and mortality. The STEP trial investigated the ideal systolic BP target in older patients to reduce cardiovascular risk [17]. It found that treatment with a systolic BP target of 110 to <130 mmHg resulted in lower incidence of cardiovascular events compared to standard treatment with a target of 130 to <150 mmHg. However, since this trial was conducted in population of Chinese hypertensive patients aged 60–80, its generalizability to a younger population is unclear.

**Table 1.** Classification of hypertension based on office blood pressure (BP) measurement.

Category	Systolic (mmHg)	and/or	Diastolic (mmHg)
American College of Cardiology/American Heart Association (2017)			
Normal	<120	and	<80
Elevated	120–129	and	<80
Stage 1 Hypertension	130–139	or	80-89
Stage 2 Hypertension	≥140	or	≥90
European Society of Cardiology/European Society of Hypertension (2018)			
Optimal	<120	and	<80
Normal	120–129	and/or	80-84
High-Normal	130–139	and/or	85-89
Grade 1 Hypertension	140–159	and/or	90-99
Grade 2 Hypertension	160–179	and/or	100-109
Grade 3 Hypertension	≥180	and/or	≥110
Isolated Systolic Hypertension	≥140	and	<90

Despite differences in the definition of hypertension, there is a lot of congruity between the ACC/AHA and ESC/ESH guidelines. Both guidelines, in addition to the 2020 Hypertension Canada guidelines [18], place a strong emphasis on measurement accuracy and are consistent with the approach of BP measurement. The outlined methodology is similar to that used in the ACCORD [19] and SPRINT [20] trials: the patient must be seated in a quiet area for five minutes before a measurement is taken, their feet must be flat on the ground and their back firmly supported, and the appropriate size of cuff must be used. Furthermore, all guidelines stress the importance of repeated readings and out-of-office monitoring in conjunction with automated office BP measurement [1,13,18,21,22]. These are used to rule out masked and white coat hypertension which lead to the under- or over- diagnosis of hypertension, respectively [21].

Out-of-office BP measurements include home BP monitoring (HBPM) which is now very commonly used among hypertensive patients [22]. HBPM has been shown to improve patient motivation for self-care as well as to help patients increase their adherence to antihypertensive medications [23]. Patients are encouraged to take at least two readings, one minute apart in the morning before taking their medications and in the evening before supper. BP measurements are averaged every 2 to 4 weeks to assess the effects of antihypertensive treatment [1].

Out-of-office BP measurements also include ambulatory BP monitoring (ABPM) [22]. Typically, the BP monitor inflates once every half an hour during the day and once an hour when the patient is asleep, allowing for detection of changes in circadian BP [24]. This is a valuable tool for measuring BP variation both day and night in patients with chronic kidney disease who exhibit altered diurnal variation in BP [25,26]. Furthermore, higher 24-h and nighttime BP readings have been associated with greater risks of deaths and composite cardiovascular outcomes, including nonfatal coronary events, heart failure, or stroke [27]. ABPM, therefore, is superior to office-based BP measurement in predicting chronic kidney disease progression and cardiovascular risk [26,28,29]. The identification of sleep disorders is also important as patients with obstructive sleep apnea have increased sympathetic activity and frequently resistant hypertension. A meta-analysis of 1904 participants with obstructive sleep apnea and hypertension found that the use of continuous positive airway pressure significantly reduced 24-h systolic BP (−5.01 mmHg) and 24-h diastolic BP (−3.30 mmHg) [30].

## Treatment goals

Due to differences in the definition of hypertension, guidelines consequently differ in BP targets (Table 2). The ACC/AHA guidelines set a universal BP goal of <130/80 mmHg, arguing that the ‘one size fits all’ strategy simplifies decisions regarding therapy [1]. In contrast, the ESC/ESH guidelines generally recommend an initial target of <140/90 mmHg and close to 130/80 mmHg, with lower targets individualized on the basis of treatment tolerance and adherence [13]. Similarly, the 2020 Hypertension Canada and the 2021 World Health Organization (WHO) guidelines both recommend a target BP goal of <140/90 mmHg in all patients without comorbidities, with lower thresholds in patients with a high risk of CVD, diabetes mellitus, and chronic kidney disease [18,31].

**Table 2.** Blood pressure targets in hypertensive patients according to clinical conditions.

Patient Population	Systolic (mmHg)	Diastolic (mmHg)
American College of Cardiology/American Heart Association (2017)		
All	<130	<80
European Society of Cardiology/European Society of Hypertension (2018)		
Aged ≥65 years	130–139	70–79
Diabetes	Close to 130 (or lower if tolerated)	70–79
Coronary artery disease	Close to 130 (or lower if tolerated)	70–79
Chronic kidney disease	130–139	70–79
All others	120–129	70–79
Hypertension Canada (2020)		
Low risk (no target organ damage or cardiovascular risk factors)	<140	<90
High risk of cardiovascular disease	<120	–
Diabetes mellitus	<130	<80
All others	<140	<90
World Health Organization (WHO 2021)		
No comorbidities	<140	<90
Known cardiovascular disease	<130	–
High risk (diabetes mellitus, chronic kidney disease, high risk of cardiovascular disease)	<130	–

## Treatment strategies

### Non-pharmacological management

Lifestyle modification is seen as the cornerstone for essential hypertension prevention and treatment. High BP is often related to unhealthy dietary habits, lack of physical activity, and/or high alcohol intake. Thus, weight loss in adults who are overweight/obese, adherence to a healthy diet, reduction in sodium intake, enhancement in potassium intake, increased physical activity, moderation in alcohol consumption, and smoking cessation are all prescribed prior to initiation of antihypertensive medication in stage I patients [1,13,18]. Effective lifestyle changes may be sufficient to delay or prevent the need for pharmacological therapy or augment its effects when used in conjunction [13].

#### Weight loss

There is a continuous almost-linear relationship between body mass index (BMI) and hypertension, with no evidence of a threshold [32,33]. Risk estimates from the Framingham Heart Study suggest that approximately 65–80% of essential hypertension can be ascribed to excess weight gain, particularly increased visceral adiposity [34]. Studies have shown that a loss of 6–8% in body weight can reduce systolic BP and diastolic BP by more than 5 and 4 mmHg, respectively [35], and weight loss of 10 kg may reduce systolic BP by 5–20 mmHg [36]. It is recommended that weight loss strategies employ a multidisciplinary approach which combines dietary education, regular physical activity, and behavioral intervention [37,38].

#### Physical activity

The antihypertensive effects of exercise training on resting and ambulatory BP have been consistently shown in many trials over the past two decades [39–41]. A meta-analysis of 5223 participants showed that moderate to high intensity aerobic exercise (<210 min/week) reduced resting systolic and diastolic BP by 8.3 and 5.2 mmHg, respectively, in adults with hypertension [39]. Another meta-analysis showed that regardless of intensity, frequency, and duration, aerobic exercise reduced around 4 and 3 mmHg of 24-h ambulatory systolic and diastolic BP, respectively [41]. Dynamic resistance training should be considered as an important supplement to aerobic training as it elicits both antihypertensive and neuromuscular benefits, such as increases in strength, power, and muscle mass [42]. A meta-analysis showed that moderate-intensity dynamic resistance training (65–75% of 1 repetition max, ∼3 days/week) reduced resting systolic and diastolic BP by approximately 6 and 5 mmHg, respectively, in adults with hypertension [40]. A network analysis comparing exercise treatments with antihypertensive medications found no detectable differences in the systolic BP-lowering effects of diuretics, angiotensin-converting enzyme inhibitors, angiotensin II receptor blockers, calcium channel blockers and beta-blockers when compared to endurance or dynamic resistance training in hypertensive participants [43].

As such, current hypertension guidelines all recommend moderate-intensity aerobic training complimented with resistance training, with variations in frequency intensity, and duration of sessions, for hypertensive adults (Table 3) [1,13,18]. There is no difference in the magnitude of BP reduction between moderate-intensity continuous training (MICT) and high-intensity interval training (HIIT) [44]. HIIT, however, improves vascular function and general cardiorespiratory fitness to a greater extent that MICT, and can be considered as an alternative approach to the traditional recommendation of MICT to hypertensive patients [44].

**Table 3.** Professional recommendations of physical activity for adults with hypertension.

	ACC/AHA 2017	ESC/ESH 2018	Hypertension Canada 2020
Frequency	–	5–7 days/week	4–7 days/week
Intensity	65%–75% heart rate reserve	Moderate	Moderate
Time	90–150 min/week	30 min/session	30-60 min/session
Type of training	Aerobic	Aerobic	Aerobic
Complementary training	Resistance training (90-–150 min/week; 50%–80% 1 rep maximum; 6 exercises, 3 sets/exercise, 10 repetitions/set)	Resistance training (2–3 days/week)	Resistance training (SBP/DBP of 140–159/90–99 mmHg)

#### Diet

The most established diets for hypertensive patients include the Dietary Approaches to Stop Hypertension (DASH) diet and the Mediterranean diet due to their robust BP-lowering effects [45,46]. In a meta-analysis of dietary pattern interventions, the DASH diet showed significant effects on systolic (−7.6 mmHg) and diastolic (−4.2 mmHg) BP, with higher reductions seen in patients with higher baseline BP [47]. Similarly, the Mediterranean diet has shown beneficial effects on systolic (−3.0 mmHg) and diastolic (−2.0 mmHg) BP, in addition to reductions in body weight (−1.8 kg) and BMI (−0.6 kg/m^2^) [48]. These two dietary patterns are rich in fruits, vegetables, legumes, nuts and seeds, moderate in fish, seafood, poultry and dairy, and low in red or processed meats and sweets [49].

Independently of dietary pattern, many trials have shown significant BP-lowering effects of sodium restriction [50,51]. The global usual intake of sodium is anywhere between 3.5–5.5 g per day, corresponding to 9–12 g of salt per day [13]. The Canadian and European guidelines both recommend limiting sodium intake to 2 g (or 5 g of salt) per day for individuals with hypertension [13,18]. American guidelines note an optimal goal of less than 1.5 g per day but advise to aim for at least a 1 mg daily reduction in most adults [1]. Most hypertension guidelines acknowledge the benefit of increased potassium intake on BP with only the 2017 AHA/ACC guidelines providing a recommended dose of 3500–5000 mg per day [1].

#### Alcohol

Due to its detrimental effect on cardiovascular health, most guidelines, including the International Society of Hypertension (ISH), ESC/ESH, ACC/AHA and Hypertension Canada, suggest that no alcohol is the safe threshold for alcohol consumption [1,13,18,52]. The 2020 Hypertension Canada guidelines recommend that both healthy and hypertensive adults should abstain from or limit alcohol consumption to less than 2 drinks a day (the equivalent of 17.2 mL of ethanol or approximately 44 mL of 40% spirits, 355 mL of 5% beer, or 148 mL of 12% wine) [18]. The 2017 AHA/ACC guidelines advise on moderation to two daily drinks for men and one daily drink from women [1]. Similarly, the 2018 ESC/ESH guidelines recommend limiting alcohol consumption to 14 weekly units for men and 8 weekly units for women (1 unit is equal to 125 mL of wine or 250 mL of beer) [13]. Additionally, European guidelines encourage alcohol-free days during the week and avoidance of binge drinking [13].

#### Smoking

Cigarette smoking has adverse effects on BP and is a major risk factor for CVD [53]. Therefore, it is important to establish a patient’s history regarding tobacco use, update their status on a regular basis, and advise on smoking cessation to hypertensive smokers. The 2017 Canadian and 2018 European guidelines both recommend the use of pharmacological measures (e.g. varenicline, bupropion, nicotine replacement therapy) in combination with behavioral intervention for smoking cessation [1,13]. The 2020 ISH guidelines advise on referral to smoking cessation programs [52].

Electronic nicotine delivery devices, or e-cigarettes, are increasingly gaining popularity, especially among youth and former cigarette smokers. Given their novelty, knowledge on the long-term health consequences of e-cigarettes remains limited. Several small studies assessing the effects of e-cigarettes on cardiovascular risk factors report significant acute increases in both systolic and diastolic BP, similar to those observed with the use of traditional cigarettes [54–56]. A 2020 umbrella review of 183 studies corroborates the adverse effect of e-cigarettes on BP management but highlights that the detrimental impact of e-cigarettes is of lesser magnitude than that of traditional cigarettes [57]. Another cross-sectional study, assessing the association of smoking and e-cigarette use to self-reported diagnosed hypertension, shows that current vaping (aOR= 1.31) and current smoking (aOR = 1.27) are both associated with similar odds of hypertension [58]. Altogether, these findings raise concerns about the harms of chronic e-cigarette use and necessitate further investigation into their long-term cardiovascular impact.

### Pharmacological management

The BP thresholds for initiation of antihypertensive therapy vary across guidelines, and are contingent on the grade of hypertension, risk of cardiovascular disease, and presence of comorbidities (Table 4). There are five major classes of first-line antihypertensive medications: thiazide/thiazide-like diuretics, angiotensin-converting enzyme (ACE) inhibitors, angiotensin II receptor blockers (ARB), calcium channel blockers (CCB) and beta-blockers [1]. Although beta-blockers are maintained as first line agents in Canadian guidelines [18], most guidelines restrict them to compelling indications, such as angina, post myocardial infarction, arrythmia, heart failure with reduced ejection fraction, or as an alternative to an ACE inhibitor or ARB in women of child-bearing potential [1,13].

**Table 4.** Blood pressure thresholds for initiation of antihypertensive therapy.

Patient Population	Systolic (mmHg)	Diastolic (mmHg)
American College of Cardiology/American Heart Association (2017)		
High risk of cardiovascular disease	130–139	80–89
All others	≥140	≥90
European Society of Cardiology/European Society of Hypertension (2018)		
Aged 18-79 years	≥140	≥90
Aged ≥80 years	≥160	≥90
Hypertension Canada (2020)		
Low risk (no target organ damage or cardiovascular risk factors)	≥160	≥100
High risk of cardiovascular disease	≥130	–
Diabetes mellitus	≥130	≥80
All others	≥140	≥90
World Health Organization (2021)		
No comorbidities	≥140	≥90
Known cardiovascular disease	≥130	–
High risk (diabetes mellitus, chronic kidney disease, high risk of cardiovascular disease)	130-139	–

**Figure 1.**
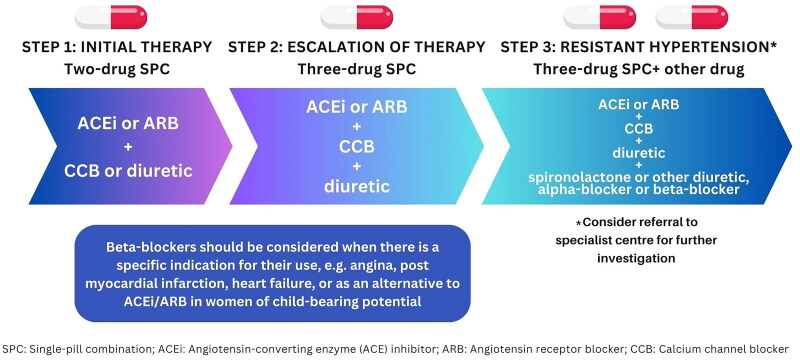
General treatment algorithm for patients with high blood pressure and no compelling indications.

Single-pill combinations are highly favored since they have been shown to improve patient adherence and increase the rate of BP control by targeting complimentary mechanisms of first-line antihypertensive agents [59,60]. The TRIUMPH and QUARTET trials showed that initiating treatment with a fixed low-dose triple or quadruple antihypertensive combination achieved and maintained greater BP reduction compared to usual care or standard dose monotherapy [61,62]. Recommended single-pill combinations are those in which an ACE inhibitor or ARB is combined with a CCB or a thiazide/thiazide-like diuretic. Typically, triple therapy is initiated when patients do not achieve adequate BP control with a dual combination of antihypertensive agents and require treatment escalation. An ACE inhibitor or ARB combined with a CCB and a thiazide/thiazide-like diuretic is recommended (Figure 1). If BP remains above-goal with the concurrent use of three first-line agents at maximally tolerated doses, a diagnosis of resistant hypertension may be made [63]. In such cases, further investigation is required to assess treatment adherence, optimal dosing of antihypertensives, and treatment resistance due to secondary causes.

Other antihypertensive medications, including alpha-blockers or mineralocorticoid receptor agonists, are no longer recommended for the routine treatment of hypertension, and are primarily reserved as add-on therapy in cases of resistant hypertension where all other treatment options have failed [13]. Spironolactone has been shown to be the most effective add-on agent in this patient population and is preferred by all guidelines as a fourth-line agent [1,13,18,64].

It is important to note that when treating hypertensive patients, therapy needs to be individualized according to their comorbidities, contraindications, and tolerances. Common comorbidities include diabetes mellitus, coronary artery disease, heart failure, atrial fibrillation, and chronic kidney disease [65]. In these cases, the choice of first-line antihypertensive agents and their doses vary.

#### Diuretics

Thiazide and thiazide-like diuretics decrease intravascular volume by promoting natriuresis and diuresis *via* blockage of the sodium-chloride channel in the distal convoluted tubule [66]. They are widely used for pharmacological treatment due to their robust effectiveness in reducing BP, favorable safety profile, and low cost. There are number of different thiazides that are available for the treatment of hypertension (Table 5). Current guidelines state that long-acting (thiazide-like) diuretics are preferred over shorter-acting (thiazide) diuretics based on their greater effectiveness in reducing the risk of cardiovascular events (12%, *p* = 0.049) and heart failure (21%, *p* = 0.023) [67]. Additionally, in contrast to thiazide diuretics, thiazide-like diuretics have also been shown to reduce coronary events and all-cause mortality [67]. A recent trial of 13,523 patients, however, showed no difference in major cardiovascular outcome events or non–cancer-related deaths after treatment with chlorthalidone or hydrochlorothiazide, bringing the superiority of thiazide-like diuretics into question [68]. Nonetheless, these results were met with controversy due to the design and inclusion criteria of the trial. Some critiques include the confounding effect of hypokalemia which may have influenced cardiosvascular outcomes as well as the lower-than-usual doses of chlorthalidone used in the trial compared to other major trials establishing the benefits of chlorthalidone therapy [69].

**Table 5.** Summary of first-line antihypertensive agents.

Name	Starting (mg/day)	Maintenance (mg/day)	Maximum (mg/day)	Additional Information	Taken with food?
Thiazide Diuretics					
Bendroflumethiazide	5	5	5	–	No
Chlorothiazide	500–1000	500–1500	2000	May be given in 1–2 divided doses	No
Chlorthalidone	12.5–25	12.5–100	100	Doses above 50 mg/day are rarely used	Yes
Hydrochlorothiazide	12.5 (if taken with other antihypertensives) or 25	25–50	50	May be given in 1–2 divided doses; Doses above 50 mg/day are rarely used	No
Indapamide	1.25	1.25–5	5	-	No
Metolazone	2.5–5	2.5–5	20	-	No
Loop Diuretics					
Bumetanide	0.5–2	-	10	2^nd^ and 3^rd^ dose may be given at intervals of 4 − 5 h up to max of 10 mg/day	No
Furosemide	40 BID	20–80	80	Decreased absorption when taken with food	Take on an empty stomach
Torsemide	5	5–10	10	-	No
Angiotensin-Converting Enzyme Inhibitors					
Benazepril	5 (if taken with a diuretic) or 10	20–40	80	May give in 1–2 divided doses	No
Captopril	25 BID-TID	25–150 BID-TID	450	Increase dose at 1–2-week intervals	1 h before meals
Enalapril	2.5 (if taken with a diuretic) or 5	10–40	40	May give in 1–2 divided doses	No
Fosinopril	10	20–40	80	May give in 1–2 divided doses	No
Lisinopril	10	20–40	40	Lower starting doses in patients taking diuretics	No
Moexipril	7.5	7.5–30	60	Lower starting doses in patients taking diuretics; May give in 1–2 divided doses	1 h before meals
Perindopril	4	4–8	16	Lower starting doses in patients taking diuretics or elderly; May give in 1–2 divided doses	No
Quinapril	10-20	20–80	80	Lower starting doses in patients taking diuretics; May give in 1–2 divided doses	No
Ramipril	2.5	2.5–20	20	May give in 1–2 divided doses	No
Trandolapril	1 (nonblack) 2 (black)	2–4	8	Lower starting doses in patients taking diuretics; May give in 1–2 divided doses	No
Angiotensin II Receptor Blockers					
Azilsartan	40–80	40–80	80	Lower starting dose in patients taking diuretics	No
Candesartan	8–16	8–32	32	Lower starting dose in patients taking diuretics; May be given in 1-2 divided doses	No
Irbesartan	150	150–300	300	Lower starting dose in patients taking diuretics	No
Losartan	50	25–100	100	Lower starting dose in patients taking diuretics; May take 3-6 weeks for max effect	No
Olmesartan	20	20–40	40	Lower starting dose in patients taking diuretics	No
Telmisartan	40	20–80	80	Lower starting dose in patients taking diuretics;	No
Valsartan	80–160	80–320	320	Lower starting dose in patients taking diuretics	No
Dihydropyridine Calcium Channel Blockers					
Amlodipine	2.5 (fragile, elderly, or hepatic insufficiency) or 5	5–10	10	Increase dose at intervals of 7–14 days	No
Felodipine	2.5 (elderly or liver disease) or 5	2.5–10	10	Do not crush, cut, or chew tablet	Take without food or a light meal
Isradipine	2.5 BID	2.5–10 BID	10 BID	Increase dose in increments of 5 mg/day at 2–4 week intervals. Max response seen in 2–4 weeks	No
Nicardipine	20 TID	20–40 TID	40 TID	Increase dose at intervals of ≥ 3 days	No
Nifedipine ER	30	30–60	120	Titrate dose over 7–14 days; Do not cut, crush, or divide tablet	Take on empty stomach; Do not take with grapefruit juice
Nisoldipine ER	17–20	17–40	40	Increase dose at intervals of ≥ 1 week; Do not cut, crush, or divide tablet	Take on empty stomach; Do not take with grapefruit juice
Lercanidipine	10	10	20	–	Take at least 15 min before food
Non-Dihydropyridine Calcium Channel Blockers					
Diltiazem ER	120–240	240–360	360	Increase dose at 14 day intervals; available in capsule or tablet; do not crush, cut, or chew	Can take with or without food
Verapamil IR	40 TID (elderly or small patients) or 80 TID	80–120 TID	360	Blood pressure effect seen within first week of therapy	No
Verapamil ER	120 (elderly or small patients) or 180	180–480	480	Blood pressure effect seen within first week of therapy; Daily doses > 240 mg should be given in 2 divided dose	Yes – capsule can be opened and sprinkled on applesauce
Beta-Blockers					
Atenolol	50	50–100	100	Increase dose at intervals of 1–2 weeks	No
Bisoprolol	5	5–20	20		No
Carvedilol IR	6.25 BID	6.25–25 BID	25 BID	Increase dose at 7–14 days	Yes
Carvedilol ER	20	20–80	80	Increase dose at 7–14 days; Capsule may be opened and sprinkled over applesauce	Yes
Labetalol	100 BID	200–400 BID	1200–2400	Increase dose in increments of 100 mg/dose at intervals of 2–3 days	Yes
Metoprolol Succinate ER	25–100	100-400	400	Increase dose at intervals of 1 week	Yes
Metoprolol Tartrate IR	100	100–450	450	May give in 1–2 divided doses; increase dose at intervals of 1 week	Yes
Nadolol	40	40–80	320	-	No
Nebivolol	5	5–40	40	Increase dose at intervals of 2 weeks	No
Pindolol	5 BID	5–30 BID	60	Increase dose in increments of 10 mg/day every 2–4 weeks	No
Propranolol IR	40 BID	60–120 BID	640	-	No
Propranolol ER (Inderal LA)	80	120–160	640	Capsule, extended release	No
Propranolol ER (Innopran XL)	80 qHS	80–120 qHS	120	Capsule, extended release; Take consistently either on an empty stomach or with food	Yes or No
Timolol	10 BID	10–20 BID	30 BID	Increase dose at intervals of ≥ 7 days	No

Adverse effects of thiazides include the following: hypokalemia, hyponatremia, metabolic alkalosis, hypercalcemia, hyperglycemia, hyperuricemia, hyperlipidemia, and sulfonamide allergy [66]. These effects stem from the ionic imbalance caused by initial sodium loss in the distal convoluted tubule. Thiazide may also increase the risk of developing acute pancreatitis [70]. As such, clinicians should closely monitor for electrolyte abnormalities and symptoms of acute pancreatitis. A recent network meta-analysis involving 58,807 participants showed that thiazides combined with potassium-sparing diuretics increased BP-lowering efficacy compared with thiazides alone, while minimizing hypokalemia and hyperglycemia [71]. These findings suggest that the combination of thiazide and potassium-sparing diuretics should be considered more frequently in the management of hypertension.

Current guidelines state that thiazides and thiazide-like agents are less effective antihypertensive agents in patients with a reduced glomerular filtration rate (estimated glomerular filtration rate <45 mL/min) and become ineffective when the estimated glomerular filtration rate is <30 mL/min [1,13,72]. In these cases, the guidelines recommend loop diuretics such as furosemide or torsemide over thiazides for their antihypertensive effects (Table 5) [1]. However, the CLICK trial as well as a recent meta-analysis suggest that thiazide and thiazide-like diuretics maintain their effectiveness in lowering BP in patients with advanced chronic kidney disease [72,73]. Loop diuretics function by inhibiting the reabsorption of sodium and chloride at the apical membrane of the thick ascending limb of the loop of Henle [74]. Like thiazides, adverse effects for loop diuretics occur from electrolyte imbalance secondary to the diuresis effects which should be closely monitored.

#### Renin-angiotensin-aldosterone system inhibitors

ACE inhibitors and ARBs both act on the renin-angiotensin-aldosterone system (RAAS) which mediates BP through the regulation of vascular tone, and sodium and fluid homeostasis [75]. Although ACE inhibitors and ARBs do not differ in effectiveness, ARBs have shown to present a better safety profile [76]. There are a large number of ACE inhibitors that are available for the treatment of hypertension (Table 5) [77]. Clinicians should be aware about potential side effects associated with ACE inhibitors include angioedema, hyperkalemia, elevated blood urea nitrogen, creatinine increase, dizziness, and syncope. Monitoring for these effects may be advisable, particularly in older patients or after dosage adjustments [78–80]. Importantly, a dry cough may also develop in approximately 10% of patients treated with ACE inhibitors, half of which will ultimately have to discontinue use [81,82]. This is the most common adverse effect of ACE inhibitors which may occur months or even a year after the institution of therapy. The cough will usually resolve within a few days after withdrawal of treatment [82].

In contrast, ARBs are generally well tolerated and are associated with significantly lower treatment discontinuation rates for adverse events than those of all other antihypertensive therapies [83]. As such, they are suggested as an alternative to patients who cannot tolerate ACE inhibitor therapy due to an induced cough or angioneurotic edema (Table 5). Of note, ACE inhibitors and ARBs should not be used in combination [84]. Furthermore, both ACE inhibitors and ARBs should be used with caution in patients with abnormal renal function, aortic valve stenosis, hypovolemia, or bilateral renal artery stenosis. They are contraindicated in pregnancy, and in patients with hyperkalemia (potassium >5.5 mmol/L). Recent guidelines also recommend discontinuation of RAAS inhibitors in women who are considering pregnancy, and caution in women of child-bearing potential who are not using reliable contraception.

There is conflicting evidence on the efficacy of ACE inhibitors and ARBs in Black hypertensive patients [85,86]. Several proposed pathophysiologic mechanisms for decreased efficacy have been advanced, including ethnic differences in metabolism and renin angiotensin aldosterone system, lack of genetic variants in cytochrome P450 2C9 which metabolizes some ARBS, and naturally lower renin levels due to baseline sodium retention [85]. There is also a higher prevalence of ACE inhibitor-induced angioedema in people of black African origin [85]. As such, an ARB is preferred over an ACE inhibitor for antihypertensive treatment in these patients. An ACE inhibitor or ARB may also be less effective in older hypertensive patients due to their decreased plasma renin levels [87]. Therefore, measuring renin levels prior to pharmacotherapy initiation may help individualize treatments in both Black and elderly populations.

#### Calcium channel blockers

CCBs function by blocking the inward movement of calcium binding to voltage-gated calcium channels in the heart and vascular smooth muscle [88]. They can be divided into two major classes based on their primary physiologic effects: dihydropyridines and non-dihydropyridines [89]. Dihydropyridine CCBs are vascular selective and have antihypertensive properties, predominately affecting peripheral vasodilatation. Contrarily, non-dihydropyridine CCBs are myocardial selective and have antiarrhythmic properties. Both classes of CCBs are used for the treatment of hypertension (Table 5) with similar effectiveness as other major drug classes on BP, major cardiovascular events, and mortality outcomes [90,91]. Additionally, CCBs are superior to other drugs for the prevention of stroke, but inferior for the prevention of heart failure [90,91].

In general, long-acting dihydropyridines (e.g. amlodipine) are preferred over intermediate-acting (e.g. nicardipine) or short-acting (e.g. nifedipine) dihydropyridines [1,18] to provide greater cardiovascular protection [92] and to simplify dosing to once-daily. Common adverse events in dihydropyridines are related to their vasodilation effects, including dizziness, facial flushing, headaches, and peripheral edema [93]. Dihydropyridines are contraindicated in patients with severe stenotic heart valve defects and hypertrophic obstructive cardiomyopathy.

Non-dihydropyridines, which include verapamil and diltiazem, may cause constipation, worsening cardiac output, and bradycardia [94]. As such, they are contraindicated in patients with heart failure with reduced ejection fraction, second or third-degree AV blockade, sick sinus syndrome, and Wolff-Parkinson-White syndrome. Furthermore, non-dihydropyridines should not be combined with beta-blockers because they can enhance the negative inotropic, chronotropic, and dromotropic effects of beta-blockers [95].

#### Beta-blockers

Beta-blockers are a class of medication which mediate cardiac activity, control various aspects of metabolic activity and induce smooth muscle relaxation [96]. Due to their effect, they lead to low cardiac output, bradycardia, higher total peripheral resistance, reduced renal blood flow and glomerular filtration rate, and low plasma renin activity. As such, beta-blockers are not recommended as first-line therapy for hypertension in high-risk patients or patients over the age of 60 unless they have comorbid diseases which necessitate beta-blocker need, such as, heart failure or ischemic heart disease [97,98].

Most beta-blockers are dosed at least twice per day while long-acting beta-blockers, such as metoprolol succinate, typically include once-daily dosing (Table 5). Labetalol is often used intravenously in patients with hypertensive crises but can also be used as an oral medication. It is not typically used as a first-line therapy for hypertension except in pregnant women [1,13,18].

All beta-blockers, especially in patients with cardiac risk factors, carry a risk of atrioventricular conduction disorders [96]. As such, clinicians should measure the heart rate of patients using these medications at each visit. Patients may also be encouraged to monitor and record their heart rates at home using BP monitors or wearable devices as needed.

Since beta receptors are found all over the body and induce a broad range of physiologic effects, their blockade may lead to many adverse effects. Some commonly reported adverse effects include fatigue, dizziness, nausea, and constipation [96]. Furthermore, beta-blockers should be used with caution in patients with asthma or chronic obstructive pulmonary disorder.

### Pregnancy and lactation

Pregnant and lactating patients require special attention, however evidence for this population is lacking and non-contemporaneous (Table 6) [99,100]. First-line oral medications that are commonly used in pregnancy include labetalol, methyldopa, and long-acting nifedipine [18,99,100]. Other oral beta-blockers, such as acebutolol, metoprolol, pindolol and propranolol, are also considered safe [18]. Second-line agents that can be used in pregnancy include clonidine, hydralazine, and thiazide diuretics. ACE inhibitors and ARBs should be avoided as they are associated with an increased risk of fetal malformations, particularly fetal renal damage [101,102]. For lactating women, and up to 6 weeks postpartum, labetalol, methyldopa, long-acting nifedipine, enalapril, and captopril are commonly used [18]. However, some guidelines suggest avoiding methyldopa due to its increased risk of postpartum depression [13,52,103].

**Table 6.** Summary of antihypertensive agents used in pregnancy.

Drug	Class	Dose	FDA Risk	Additional Information
**First-line agents**				
Methyldopa	Central alpha agonist	500–3000 mg/day in 2 divided doses	B	Drug of choice according to National High Blood Pressure Education Program
Labetalol	Combined alpha and beta blocker	200–1200 mg/day in 2–3 divided doses	C	May be associated with fetal growth restriction
Long acting Nifedipine	Calcium channel blocker	30–120 mg/day	C	May inhibit labor and have synergistic BP-lowering action with magnesium
**Second-line agents**				
Clonidine	Central alpha agonist	0.1–0.6 mg/day in 2 divided doses	C	Similar efficacy to methyldopa
Hydralazine	Peripheral vasodilator	50–300 mg/day in 2–4 divided doses	C	Useful in combination with sympatholytic agent; may cause neonatal thrombocytopenia
Hydrochlorothiazide	Thiazide diuretic	12.5–25 mg/day	C	May cause volume contraction and electrolyte abnormalities; May be useful in combination with methyldopa and vasodilator to mitigate compensatory fluid retention

### Novel therapies

#### Drug therapies

Novel therapies targeting specific pathways involved in BP regulation are now being investigated for the management of hypertension. Zilebesiran is a small-interfering RNA which inhibits hepatic angiotensinogen synthesis, subsequently lowering BP [104]. A phase I study demonstrated that a single subcutaneous dose of zilebesiran reduced systolic BP by 10 mmHg and diastolic BP by 5 mmHg in 8 weeks [105]. Neprilysin inhibitors, in combination with angiotensin receptor blockers, enhance natriuretic peptide levels, promoting vasodilation and diuresis. In a meta-analysis of 6028 participants, the dual agent sacubitril/valsartan was more effective (−4.62 mmHg systolic BP, −2.13 mmHg diastolic BP) than an ARB in BP reduction among hypertensive patients [106]. Aldosterone synthase inhibitors and endothelin receptor agonists have shown promising results in phase II and phase III trials of BP reduction in patients with resistant hypertension [107,108]. Glucagon-like peptide-1 receptor agonists, previously used for glycemic control in patients with diabetes, are now being investigated for their weight loss effects which consequentially lead to BP reduction [109–112]. In a meta-analysis of 4,567 patients, semaglutide induced a body weight loss of −10.09%, and reduced systolic BP by −5.10 mmHg compared to placebo [113]. SGLT-2 inhibitors, also designed for diabetes, have shown significant BP reduction. A meta-analysis of 9,913 participants showed a systolic BP reduction of −5.06 mmHg and a diastolic BP reduction of −2.39 mmHg compared to placebo [114].

#### Interventional therapies

Recent advancements have introduced novel approaches to effectively manage hypertension beyond conventional pharmacological therapy. Clinicians should be aware of these options, particularly in cases of resistant hypertension. Renal denervation is a minimally invasive catheter-based procedure which disrupts the sympathetic nerves surrounding the renal arteries to achieve sustained BP reductions. Renal denervation has shown reductions of around 5 mmHg in systolic BP and 2 mmHg in diastolic BP [115]. Furthermore, recent 9-year follow up data shows robust reduction in both office and ambulatory systolic and diastolic BP in patients with resistant hypertension [116]. Baroreceptor activation therapy is a surgical technique which electrically stimulates the carotid baroreceptors to reduce sympathetic nerve activity and, subsequently, BP through an implantable device. It has also shown significant reductions of systolic BP in patients with resistant hypertension [117].

## Monitoring and follow-up

Routine tests for all hypertensive patients include hemoglobin and hematocrit, fasting blood glucose and/or HBA1c, lipid profile, serum sodium and potassium, serum creatinine with estimated glomerular filtration rate, serum uric acid, liver enzymes, urinalysis, and 12-lead electrocardiogram [18]. Regular monitoring and follow-ups are crucial when treating hypertension to assess response to treatment, detect any adverse events or complications, and adjust the treatment plan as required. Monthly office follow-ups are recommended after initiation or change in antihypertensive medication until patients reach their target BP [1,13,18]. For patients with controlled BP, follow-ups are recommended every 3–6 months [1,13,18,118]. In addition, patients who have difficulty remembering to take their medication and patients with diabetes may benefit from daily or frequent HBPM [18,119,120].

Telemedicine has shown significant potential in the management of essential hypertension by enhancing patient engagement and treatment adherence in the outpatient setting [121–123]. Telemedicine enables patients to connect with clinicians remotely for regular check-ins which not only saves time and resources but also improves access to care, especially for individuals in rural or underserved areas [121]. Telemedicine can be particularly useful for patients who require close monitoring, as it allows clinicians to assess progress without requiring in-person visits.

## Conclusion

In conclusion, hypertension is globally the leading cause of cardiovascular disease and premature death [124]. Although the classifications and definitions of hypertension vary across guidelines, there is a shared goal of utilizing evidence-based research to provide effective strategies to prevent and manage hypertension. Treatment goals and strategies must be individualized to a patient’s lifestyle, comorbidities, and preferences to minimize potential harm and increase the likelihood of long-term compliance. Lifestyle modifications are recommended before initiation of pharmacological therapy in low-moderate risk patients, and alongside pharmacological therapy in higher risk patients. Monotherapy with first-line antihypertensive agents, including diuretics, RAAS inhibitors, CCBs, and beta-blockers, is often inadequate for most hypertensive patients. As such, single-pill combinations are recommended to approve the speed, efficiency, and consistency of initial BP reduction, and long-term BP control. Although great strides have been made in North America and Europe in terms of the identification of patients with hypertension and their treatment, there is still a long way to go. Adequate identification and treatment of hypertension will substantially decrease morbidity and mortality in this population.

## Data Availability

Not applicable

## Data availability statement

Data sharing is not applicable to this article as no new data were created or analyzed in this study.
